# Synchronously diagnosed lymph nodal collision tumor of malignant melanoma and chonic lymphocytic leukemia/small lymphocytic lymphoma: case report

**DOI:** 10.1186/1746-1596-2-34

**Published:** 2007-08-30

**Authors:** Dina El Demellawy, Catherine Ross, Monalisa Sur, Salem Alowami

**Affiliations:** 1 Northern Ontario School of Medicine, Department Of Pathology and Laboratory Medicine, West Campus, Thunder Bay, Ontario, Canada; 2 McMaster University, Department Of Pathology and Molecular Medicine, Hamilton, Ontario, Canada

## Abstract

Synchronous composite tumors have been described but are uncommon. Moreover, simultaneous occurrence of synchronous tumors in the same tissue or organ is even less common. We report a case of chronic lymphocytic leukemia (CLL)/small lymphocytic lymphoma and malignant melanoma (MM) occurring synchronously in the same lymph node. Several cases of an association between cutaneous malignancies and lymphoproliferative disorders have been reported. Some of which included CLL and MM, occurring in the same patient often CLL after MM. The risk of having CLL after MM has been reported to be increased. Various genetic and environmental etiologies have been postulated, but have as yet not been proven. To our knowledge this is the first time that synchronous occurrence of these two malignant processes in the same tissue is described. In this case it is important that the melanoma was recognized in the excised lymph node, as this finding had much more critical treatment and long term survival consequences.

## Background

Synchronously diagnosed concomitant tumors are uncommon, and occurrence of collision tumors is rare. We report a case of chronic lymphocytic leukemia (CLL)/small lymphocytic lymphoma (SLL) and malignant melanoma (MM) occurring synchronously in the same lymph node. CLL and MM occurring in the same patient, often CLL after MM with a standardized incidence ratio of 2.3 [1]. Nevertheless, the relative risk of having CLL after MM has been reported to be increased (standardized incidence ratio of 2.3)[1] and the converse (i.e. melanoma) after CLL also occurs with and estimated observed to expected ratio of 2.79 [2].

Various genetic and environmental etiologies have been postulated, but have as yet not been identified. To our knowledge this is the first time that synchronously diagnosed collision tumor formed of CLL/SLL and MM is described. In this case it is important that the melanoma was recognized in the excised lymph node, as this finding had much more critical treatment and long term survival consequences.

## Case presentation

A 58 year old man presented with a right axillary mass, of 4 months duration. He was otherwise relatively well and asymptomatic. Past medical history was significant for two previous excisions of cutaneous lesions, which were diagnosed as MM involving the left and right sides of the back, nine years and two years ago respectively. The patient has no history of receiving chemo or radiotherapy. A physical examination performed at this time revealed a right axillary mass with local tenderness measuring 10.0 × 8.0 cm. The patient had been experiencing night sweats, fevers and a twenty pound weight loss. The remainder of the physical examination was significant for splenomegaly, with the spleen tip palpated 7 cm. from the left lower costal margin. No other lymphadenopathy or organomegaly was detected.

### Hematologic investigations

The peripheral blood showed a white blood cell count of 20.2 × 10^9 ^(reference range: 5–11 × 10^9^). In addition, the CBC revealed hemoglobin value of 12.6 g/dl (reference range for men: 14.0–18.0 g/dl), and platelets was 203 × 10^-6^/Liter (reference range: 150,000 to 450,000 platelets per microliter) of blood. The peripheral smear showed smudge cells.

As the patient presented with lymphadenopathy and showed leukocytosis in the peripheral blood, flow cytometry was performed on the patient's peripheral blood. This showed a small population of monoclonal B cells with Kappa light chain restriction, indicating a lymphoproliferative disorder. The cells were CD5 and CD23 positive and CD38 and FMC7 negative. This phenotype is diagnostic of CLL in the presence of the morphological features in the peripheral blood smear.

**Figure 1:**
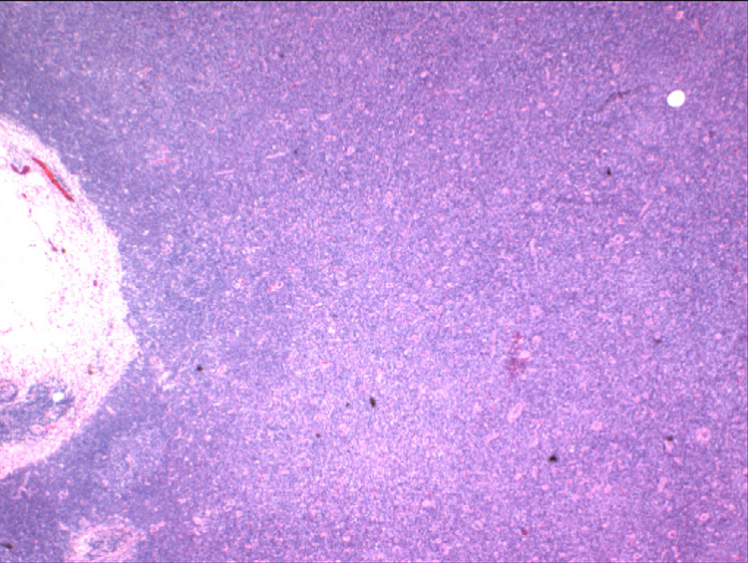
Lymph node involved with chronic lymphocytic leukemia showing architectural effacement, (HE 40×).

**Figure 2:**
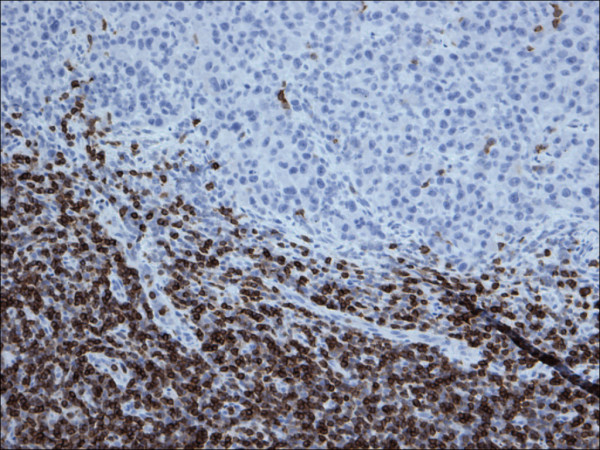
Restricted expression of CD5 within the lymphocytes in chronic lymphocytic leukemia in contrast to malignant melanoma cells, (CD5 200×).

**Figure 3:**
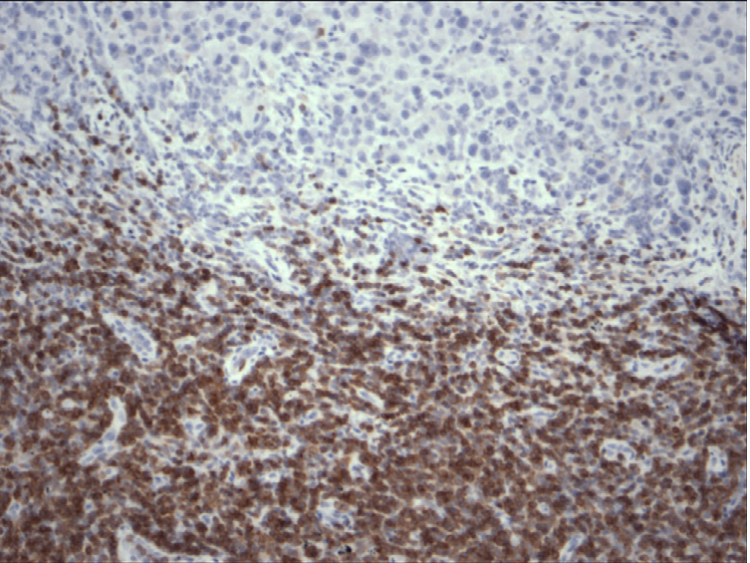
Restricted expression of Bcl-2 within the lymphocytes in chronic lymphocytic leukemia in contrast to malignant melanoma cells, (Bcl-2 200×).

**Figure 4:**
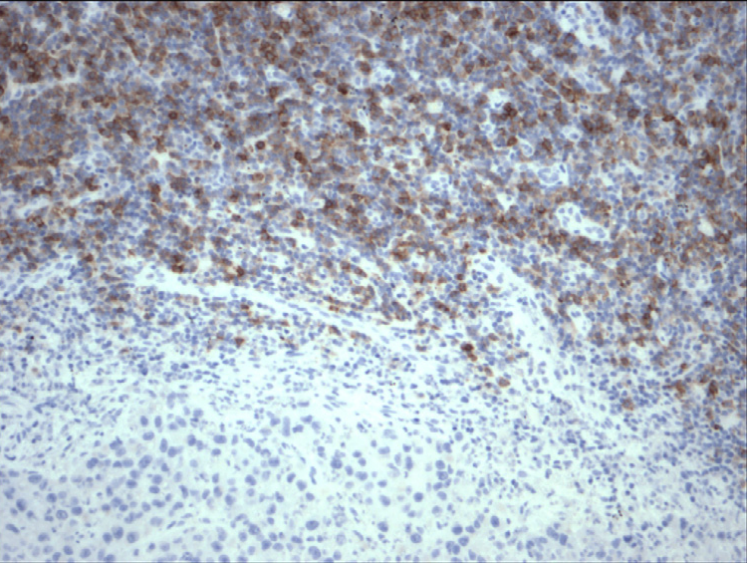
Restricted expression of CD23 within the lymphocytes in chronic lymphocytic leukemia in contrast to malignant melanoma cells, (CD23 200×).

**Figure 5:**
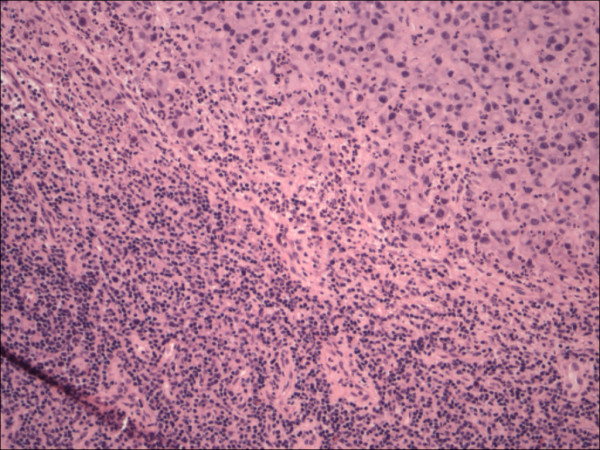
Simultaneous lymph node involvement by large epithelioid melanoma cells and small monotonous lymphocytes of chronic lymphocytic leukemia, (HE 200×).

**Figure 6:**
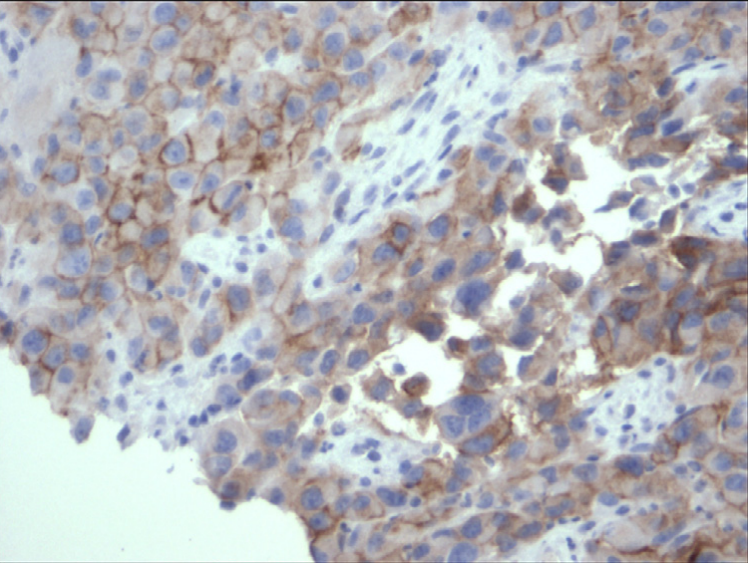
Expression of Melanoma cocktail within the cytoplasmic membranes of the malignant melanoma cells, (Melanoma cocktail 400×).

**Figure 7:**
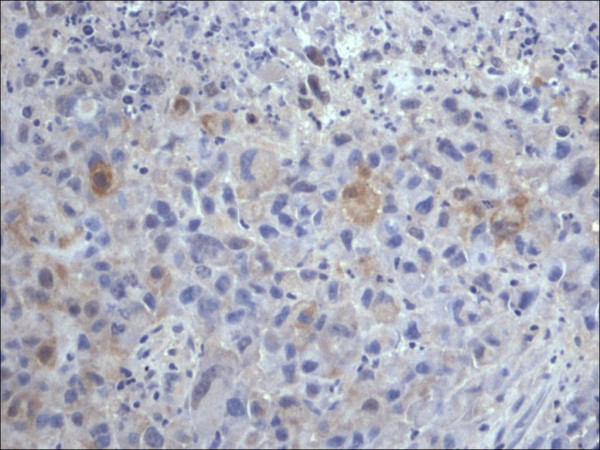
Expression of S100 within the nuclei and cytoplasm of the malignant melanoma cells, (Melanoma cocktail 400×).

Excision of the lymph node showed effacement of the normal nodal architecture by a lymphoproliferative disorder composed of small cells (fig. 1), which on immunohistochemistry [table 1] were CD20, CD5 (fig. 2), bcl-2 (fig. 3), CD23 (fig. 4) positive, CD10 and cyclin D1 negative compatible with small lymphocytic lymphoma. Only a single section showed a small metastatic focus of MM (fig. 5), measuring 1.5 mm in maximum diameter. This was of the epithelioid type that expressed MM markers, (fig. 6, 7) consistent with the previous diagnosis.

**Table 1:** The panel of immunohistochemical reagents used

Antibody	Clone	Manufacturer	Dilution
CD 20	L26	Dako (Carpinteria, CA)	1/1000
Bcl 2	124	Dako (Carpinteria, CA)	1/25
CD 5	4C7	Novocastra (Vision Biosystem, Norwell, MA)	1/50
CD 23	1B12	Novocastra (Vision Biosystem, Norwell, MA)	1/50
CD 10	56C6	Novocastra (Vision Biosystem, Norwell, MA)	1/25
Cyclin D1	SP4	MEDICORP/LAB VISION (Montreal, Quebec)	1/75
S 100	S100	Dako (Carpinteria, CA)	1/4000
Pan melanoma cocktail	HMB45, MART-1, Tyrosinase	BIOCARE MEDICAL (Concord, CA)	1/500
HMB 45	HMB 45	Dako (Carpinteria, CA)	1/200

### Clinical outcome

The patient had a re-excision of the growing axillary mass. He then had a new supraclavicular lymph node about one month later as well as persistent edema of the right arm. He received some radiation treatment at this time, after which he appeared stable for about four months. The patient then presented with shortness of breath and feeling unwell, and CT scan showed multiple nodules in the lung fields consistent with metastatic disease, at which time chemotherapy was started.

## Discussion

Synchronously diagnosed collision tumors are considered a rare occurrence.

Cases presenting as multiple primary neoplasms may occur concurrently as collision tumors or may be separated temporally. It is important to realize that the constellation of neoplasms may be more than coincidental and may reflect underlying shared etiologic factors or may be related to altered immunity [3], particularly given the immunologic derangements that are known to occur with CLL. Of course, underlying genetic susceptibilities may also play important, although as yet not well delineated roles.

In the current case, we report a composite MM and CLL involving a single lymph node in a patient with MM diagnoses known previously. However, his past medical history indicates absence of chemo or radiotherapy; that may contribute to the present evolution of CLL. CLL and MM have been reported previously as a metachronously diagnosed tumors in the English literature with MM usually a subsequent cancer in patients with CLL [4]. CLL/SLL developing after MM has also been reported. As far as we know only two synchronously diagnosed cases of CLL and MM have been reported previously in the English literature[5,3] [table 2], but none of these cases showed collision involvement of MM and CLL within the same site/organ.

**Table 2:** Reported cases of chronic lymphocytic leukemia/small lymphocytic lymphoma (CLL) and malignant melanoma (MM).

Cases reported	Age/Gender	Risk factor	Sites MM/CLL	Breslow thickness	Clark's level	Stage MM/CLL	Interval between the diagnosis of the two tumors	Follow up/duration
Schmid-Wendtner MH et al, 2002	46/F	None	Shoulder(MM)/Axillary LN (CLL)	3 mm	IV	III/I	Metachronous (MM presented first)	Not reported
Cahill RA et al, 2001	67/F	Present*	Lip (MM)/BM (CLL)	>3 mm	V	III/I	Metachronous (CLL presented first)	Well/6 months
Current case	68/M	None	Back(MM)/Axillary LN (MM & CLL)	>3 mm	III	III/II	Synchronous (MM & CLL on recurrence)	Well/2 years

* Farmer with history of exposure to ambient sunlight and carcinogenic chemicals.

• BM: Bone marrow, LN: Lymph node.

Synchronously diagnosed collision tumors of non Hodgkin's lymphomas (NHL) and MM have been reported involving the same lymph node [5-7]. Such reports include follicular, mantle and diffuse large cell lymphomas [5-7]. The current report adds to the list CLL and MM which is to the best of our knowledge is the first report of synchronously diagnosed CLL and MM involving the same node at the same time.

The association of malignant melanoma with other malignancies has been reported, including lymphoma, breast cancer, leukemia[8] and renal cell carcinoma [9]. In addition, there is a known risk of risk of second malignant neoplasms in CLL patients, including malignancies of the lung, the brain and MM [2].

Mature B cell neoplasms comprise ~4% of new cancers each year around the world [10] and they account for over 90% of lymphoid neoplasms world wide [11]. About 6.7% of non Hodgkin lymphomas are classified as SLL/CLL [11]. The majority of CLL patients are >50 years old with a median age of 65 years [12].

The median age at diagnosis of MM is 53 years as MM is now the commonest malignancy in Caucasians, between 25 and 29 years and in Caucasian men between 35–39 years [5]. The relative risk of subsequent MM for those with CLL has been estimated to be between 2.79 and 3.1 [13,14].

Patients who have MM have been ascribed a future risk of 1.9 for developing CLL [2].

There is no identifiable common cytogenetic abnormality or oncogene known between CLL and MM at this time. Risk factors for developing malignant melanoma are known to include ambient overexposure to sun light and perhaps chemical exposure in some studies. With respect to CLL, there are no proven studies to document sun light overexposure or chemical as risk factors. However, UVB present in sunlight has been suggested as a risk factor by inducing immunosuppressive effects through deficient antigen presentation and reduced T cell activity [15,16].

Composite tumors do raise the question of possible etiological links and are of academic interest from this point of view. More immediate questions include the impact of composite tumors on management and prognosis. Though CLL is eventually lethal, it usually takes a protracted and indolent course. The overall median survival of CLL is 7 years [10]. On the other hand, MM stage II and above presents a rather more significant survival threat. Most then would consider that the primary target is to manage the MM. Cytotoxic therapy, which is the mainstay of systemic therapy for lymphoma[17], has a limited efficacy in MM [5]. Although both MM and lymphoma are radiosensitive[18] this raises issues of secondary undesired consequences as radiation to areas of previous lymphadenectomy show a higher risk of subsequent lymphedema (~20%)[5].

We present a collision tumor of CLL and MM, a report to our knowledge has not been reported in the English literature. Through this case we reviewed the cases of synchronous CLL and MM and expanded on the incidence, demography, etiological correlation and management as well as prognostic implications. In this case the importance of diagnosing the small amount of malignant melanoma in the enlarged lymph node primarily occupied by CLL/SLL was of critical importance in this patient for decision making and treatment purposes, in addition to having a significant adverse impact on overall survival.

## References

[B1] McKenna DB, Stockton D, Brewster DH, Doherty VR (2003). Evidence for an association between cutaneous malignant melanoma and lymphoid malignancy: a population-based retrospective cohort study in Scotland. Br J Cancer.

[B2] Riou JP, Ariyan S, Brandow KR, Fielding LP (1995). The association between melanoma, lymphoma and other primary tumors. Arch Surg.

[B3] Schmid-Wendtner MH, Lebeau A, Sander CA, Volkenandt M, Emmerich B, Wendtner CM (2002). Lymphadenopathy detected by ultrasound examination as first diagnostic hint of chronic lymphocytic leukemia in a patient with melanoma. JEADV.

[B4] Greene MH, Hoover RN, Fraumeni JF (1978). Subsequent cancer in patients with chronic lymphocytic leukemia – a possible immunologic mechanism. J natl Cancer Inst.

[B5] Cahill R, McGreal G, Neary P, Redmond HP (2001). Synchronous high risk melanoma and lymphoid neoplasia. Melanoma Res.

[B6] Dickinson M, Wotherspoon A, Cunningham D (2006). Sub-clinical disssemination of follicular lymphoma in normal sized lymph nodes may not be detected by radiological staging: a case of disseminated follicular lymphoma.

[B7] St Peter SD, Roarke MC, Conley CR, Pockaj BA (2002). Sentinel lymph node biopsy demonstrating concomitant melanoma and mantle cell lymphoma. Surgery.

[B8] Tihan T, Filippa DA (1996). Coexistence of renal cell carcinoma and malignant lymphoma. Cancer.

[B9] Travis LB, Curtis RE, Hnakey BF, Fraumeni JF (1992). Second cancers in patients with chronic lymphocytic leukemia. J Natl Cancer Inst.

[B10] Harris NL Mature B-cell neoplasms. In tumors of hematopoietic and lymphoid tissues, World Health Organisation, 2001.

[B11] Anon (1993). A predictive model for aggressive non Hodgkin's lymphoma. The international non Hodgkin's lymphoma prognostic factors project. N Engl J Med.

[B12] Muller-Hermelink HK, Montserrat E, Catovsky D, Harris NL (2001). WHO classification of tumors: tumors of hematotopoietic and lymphoid tissues.

[B13] Adami J, Frisch M, Yuen J, Glimelius B, Melbye M (1995). Evidence of an association between non Hodgkin's lymphoma and skin cancer. BMJ.

[B14] Levi F, Randimbison L, Te VC, La Vecchia C (1996). Non Hodgkin's lymphoma, chronic lymphocytic leukemias and other skin cancers. Br J Cancer.

[B15] Streilein JW, Taylor JR, Vincek V, Kurimoto I, Shimizu T, Tie C, Golomb C (1994). Immune surveillance and sun-light induced skin cancer. Immunol Today.

[B16] Saijo S, Bucana CD, Ramirez KM, Cox PA, Kripke ML, Strickland FM (1995). Deficient antigen presentation and Ts induction are separate effects of ultraviolet radiation. Cell Immunol.

[B17] Armitage JO (1992). Chemotherapy for non Hodgkin's lymphoma. Curr Opin Oncol.

[B18] Shulamn LN, Mauch PM (1995). Current role of radiotherapy in Hodgkin's and non Hodgkin's lymphoma. Curr Opin Oncol.

